# Dysbiotic Events in Gut Microbiota: Impact on Human Health

**DOI:** 10.3390/nu6125786

**Published:** 2014-12-11

**Authors:** Serena Schippa, Maria Pia Conte

**Affiliations:** Public Health and Infectious Diseases Department, “Sapienza” University of Rome, Rome 00185, Italy; E-Mail: mariapia.conte@uniroma1.it

**Keywords:** microbiota, dysbiosis, healthy, disease

## Abstract

The human body is colonized by a large number of microbes coexisting peacefully with their host. The most colonized site is the gastrointestinal tract (GIT). More than 70% of all the microbes in the human body are in the colon. The microorganism population is 10 times larger of the total number of our somatic and germ cells. Two bacterial phyla, accounting for more than 90% of the bacterial cells, dominate the healthy adult intestine: *Firmicutes* and *Bacteroidetes*. Considerable variability in the microbiota compositions between people is found when we look at the taxonomic level of species, and strains within species. It is possible to assert that the human microbiota could be compared to a fingerprint. The microbiota acts as a barrier from pathogens, exerts important metabolic functions, and regulates inflammatory response by stimulating the immune system. Gut microbial imbalance (dysbiosis), has been linked to important human diseases such as inflammation related disorders. The present review summarizes our knowledge on the gut microbiota in a healthy context, and examines intestinal dysbiosis in inflammatory bowel disease (IBD) patients; the most frequently reported disease proven to be associated with changes in the gut microbiota.

## 1. Introduction

Despite a large number of reports indicating that changes in the microbiota composition are associated with several diseases, the definition of “healthy gut microbiota” remains unclear. The composition and roles of the bacteria that are part of this community have been intensely studied in the past few years. To understand what is the biological significance of the different microbial community associated with disease states, a “healthy” gut microbiota must be defined. Although more than 100 bacterial phyla have been described, the adult human intestinal microbiota is dominated by only two phyla, *Bacteroidetes* and *Firmicutes*, and in smaller amounts, *Proteobacteria, Verrucomicrobia, Actinobacteria, Fusobacteria* and *Cyanobacteria* [1]. Looking at the taxonomic level of bacterial species and strains, the intestinal microbiota exhibits a significant variability between people, comparable to a fingerprint. The microbiota strongly influences the development and maintenance of immune homeostasis, acts as a barrier from pathogen invasion, and provides the host with nutritional contributions [1,2]. Shifts in microbial community composition (dysbiosis) can destroy these mutualistic relationships, and influence host physiology, compromising human health status [3]. Intestinal dysbiosis has been linked with important human diseases, including autoimmune and/or autoinflammatory disorders, such as IBD, metabolic disorders, such as, obesity, type 2 diabetes [4,5], allergies [6], and neurological disorders [3,7].

Furthermore, during adult life, intestinal bacterial populations are still prone to perturbations that could lead to important consequences for our physiology, and our health status [8]. In the present review, we summarize our knowledge on the gut microbial composition in a health context, and examine intestinal dysbiosis. Our discussion will be focused on patients with IBD, an intestinal disorder extensively studied in the last years [8,9], and demonstrated to be associated with changes in the gut microbiota.

## 2. Gut Microbiota

### 2.1. Composition

All surfaces of the human body that are exposed to the external environment are colonized by microorganisms, and the gastrointestinal tract (GIT), with more than 70% of all microbes in the human body, is the most colonized organ [10]. The microbial diversity of the human gut is the result of co-evolution between microbial communities and their hosts [11] although environmental factors strongly influence the bacterial ecosystem composition [12]. More than 80% of gut bacterial species are refractory to culture-based methods [13], however, remarkable advances in DNA sequencing technologies, and other molecular techniques have allowed a more comprehensive examination of microbial communities [14]. Trillions of microbes live in the human gut. These microbes belong to all three domains of life on Earth, *Bacteria*, *Archaea* and *Eucarya*, and outnumber our own human cells, carrying about 10 million unique genes [15,16]. Microbial load in the intestines is not homogeneous “ranging from 10^1^ to 10^3^ bacteria per gram in the stomach and duodenum, progressing to 10^4^ to 10^7^ bacteria per gram in the jejunum and ileum and culminating in 10^11^ to 10^12^ cells per gram in the colon” [17], the highest recorded for any microbial habitat. Finally the mucosa associated microbiota composition significantly differs from the lumen microbiota, as well as the microbiota present near the epithelium [10,11,12,13,14,15,16,17,18]. Studies based on 16S rDNA sequencing have highlighted that only few, of the 80–100 bacterial phyla described on Earth, are detected in human gut [19]. Of the 90% of all the bacterial species detected, most belong to two bacterial phyla: *Bacteroidetes* and *Firmicutes*, with a smaller representation of bacteria belonging to *Proteobacteria* and *Actinobacteria*, indicating the existence of a strong selective pressure in the intestinal habitat [13,20]. At species and strain taxonomic level, the intestinal microbiota is highly diverse and exhibits high variability between individuals. The presence in different subjects of a “functional core” of redundant genes among different bacterial species has been recently reported [21,22]. This common functional core of genes, conserved in each subject and among different bacterial species, could reflect the factors required for the survival in the gut habitat and guarantee all the important tasks for human health such as the preservation of microbial functions. In fact, some of the functions included in such a “core of genes” are linked to the degradation of complex polysaccharides, synthesis of short-chain fatty acids (SCFAs), amino acids and vitamins. Moreover the smallest set of genes has been identified [21,22], representing the “human variable microbiome”, and depend on a combination of host-specific factors, such as genotype, physiological status, host pathologies, lifestyle, diet, and environment. Core and variable components of the human microbiome influence different aspects of human health, including nutrient responsiveness and immunity. In a recent study, the microbiota of stool samples, representing different ethnicities, countries, and continents, have been compared in order to investigate variation across the world. The authors showed the existence of three enterotypes in the human gut microbiota that vary in species, functional composition and are nation, continent or sex independent [23]. Each of these three enterotypes is characterized by the variation in the levels of one genus: enterotype 1 is dominated by *Bacteroides*, enterotype 2 is driven by *Prevotella,* and enterotype 3 is mostly enriched in *Ruminococcus.* New studies challenge these findings, suggesting that the boundaries between the enterotypes are quite thin, and the “enterotypes hypothesis” is far from being confirmed [24]. The microbiota composition undergoes a natural selection, operating at two levels [11]: (i) the “top-down level” a host selection, favors stable societies with a high degree of functional redundancy, although there are many examples of functions where the level of redundancy is really low, for example, methanogenesis is carried out by a very small number of species in the gut, and is predominantly carried out by a single species. In this case the “keystone species” (defined as a species with a central role in the gut system, the loss of which could causes dramatic loss of important human necessities) can be important [25]; and (ii) the “bottom-up level” a microbe selection, favoring microbial cells to become functionally specialized [11]. Functional redundancy in a microbiota, also known as the “insurance hypothesis” [26], confers stability ensuring the preservation of important microbial functions going against the concept of the “keystone species”. Functional specialization of the microbial cells, through genetic diversification, will enable the colonization of different ecological niches lowering bacterial competition phenomena.

### 2.2. Metabolic Functions

The gut microbiota acts as a “metabolic organ” that interacts with the human host and performs many essential functions to maintain human health status [27]. Metabolic functions of the gut microbiota allow the human host to utilize many energetic sources. The breakdown of complex indigestible dietary carbohydrates and proteins is possible thanks to the metabolic activity of the gut microbiota. Moreover, the microbiota produces vitamins, synthesizes amino acid, influences ion absorption, and is involved in the conversion of dietary polyphenolic compounds and in the bile acid biotransformation process [28,29,30]. Studies performed on the metabolic profiles of human and mice revealed that absorption, storage, and metabolism of dietary lipids can be specifically modulated by the microbiota [31]. The intestinal microbiota is able to transform potentially carcinogenic compounds, such as *N*-nitroso compounds (NOCs), and heterocyclic amines (HCAs), and to activate bioactive compounds including phytoestrogens” [32,33]. SCFAs production is an important “microbiota function”. SCFAs, mainly acetate, propionate and butyrate, positively influence intestinal epithelial cell proliferation, differentiation and have different metabolic features [34]. Butyrate is used as an energy substrate for colonocytes, and can strengthen the colonic defense barrier by inducing mucin secretion, trefoil factors, and antimicrobial peptides. Moreover butyrate exerts multiple effects, such as immune modulation cell cycle inhibition, induction of programmed cell death and cellular differentiation, in a variety of cell types [35,36]. Recently it has also been shown that butyrate is able to alter dendritic cell response to bacterial antigens, up-regulating IL-23 production [37]. Recent evidence shows that SCFAs are pivotal for the generation of Foxp3^+^ regulatory T (T_reg_) cells that play an important role in the suppression of the inflammatory response. In particular, butyrate and propionate seem to be most active in promoting T_reg_ cell differentiation. These findings clarify the relationship between microbiota and its host, and provide new therapeutic approaches able to promote intestinal homeostasis and health [38,39,40,41].

The success of medical treatments is also influenced by gut microbiota metabolic activity. An interesting study showed how the gut microbiota has the ability to inactivate drugs delivered into the intestine, with the potential to generate toxic compounds such as hydrogen sulfide (H_2_S) [42]. Hydrogen sulfide can be produced by the utilization of alimentary and endogenous compounds containing sulfur (mucin proteins), and amino acids. The gut normally converts luminal hydrogen sulfide to thiosulfate, which during inflammation is further oxidized to tetrathionate. High concentrations of H_2_S severely inhibit cytochrome 1c oxidase, blocking mitochondrial activity; H_2_S, at micromolar concentrations, however, could exert a detoxifying effect and inflammation relief [43,44]. The intestinal microbiota is also involved in the reduction of nitrate via nitrite to nitric oxide (NO) [45]. Nitric oxide (NO) has been described to have damaging effects on the energy metabolism of colonocytes, but a recent study showed that intrarectal administration of NO, in DSS-induced experimental colitis mice, has anti-inflammatory effects. Additionally, as reported in a recent publication by Korecka *et al.* [19], components of microbiota-derived metabolites could be transported via blood circulation to various organs, and potentiate multiple effects in brain (cognitive function behavior), in liver (lipid and drugs metabolisms), and in pancreas (glucose metabolism), and “at the same time, the gut brain axis can circumvent intestinal absorption, and allow the microbiome to directly affect the brain”. It appears clear from this brief discussion that an altered microbial composition may have an impact on important physiological processes.

### 2.3. Epithelium Barrier Integrity Effect

Microbiota members contribute to the maintenance of intestinal epithelium barrier integrity maintaining cell-to-cell junctions, promoting epithelial repair following injury, and playing a role in the regulation of enterocytes turnover [46]. Moreover experiments performed in germ-free mice showed a reduced number of cells per crypt [47]. Germ-free piglets display aberrant intestinal morphology with longer villi and shorter crypts probably due to a modified expression of ileal genes involved in apoptotic, and proliferative activities [48]. *Bacteroides thetaiotaomicron* species play an important role inducing the expression of the sprr2a gene encoding the small proline-rich protein II, involved in desmosome maintenance [49]. Furthermore “signaling via toll-like receptors (TLRs), mediated principally by commensal bacterial products, was shown to promote the integrity of the intestinal epithelial barrier function” [50]. The interaction between gut microbiota and TLRs, by Rakoff-Nahoum and colleagues [50], reveals a new non-immune function of these receptors. The recognition of the commensal bacteria products allows TLR to control tissue homeostasis [50].

### 2.4. Defense Barrier

The ability of microbiota to prevent pathogenic colonization, by competing for attachment sites and nutrients, and through production and secretion of antimicrobials, is termed “colonization resistance” [51,52,53]. Through this mechanism the intestinal microbiota protects itself against attacks by exogenous microorganisms. Antibiotic administration strongly decreases colonization resistance in humans. Germ-free mice infected by *Salmonella* showed a more severe gut inflammation, and mice colonized with different types of microbiota display a diverse susceptibility to pathogen infection [54]. Commensal microbiota have also been found to regulate the production of intestinal mucins by goblet cells, capable of inhibiting bacterial adhesion to intestinal epithelial cells. It has been recently reported that *Faecalibacterium prausnitzii*, can modulate the enhancing effects of *B. thetaiotaomicron* on goblet cells and on mucus production and mucin glycosylation [55]. The defense barrier of commensal microbiota could also be related to bacterial metabolic products. Volatile fatty acids, produced by anaerobic bacteria, results in toxicity for many bacterial species, and the production of bacteriocins by enterobacteria, streptococci, and anaerobic bacteria, seems to limit bacterial overgrowth.

### 2.5. Host Defense Development

The microbiota is essential for the development of a functional immune system, affecting both innate and adaptive immunities, and in promoting immune-regulation at the intestinal surface. Previous relevant studies on the involvement of microbiota on development of host defense showed that in antibiotic-treated mice the mucosa is more permeable to commensal bacteria, and less capable of post-injury repair [51,55]. After antibiotic treatment, alterations on luminal and mucosal-associated bacterial communities, and in cytokine production by mucosal CD4^+^ T lymphocytes [56,57], was shown. The critical importance of gut microbiota in the development of intestinal mucosa and the systemic immune systems can be readily appreciated from studies performed on germ-free (GF) animals [1]. Generally, germ-free mice are more susceptible to infection and have smaller Peyer’s patches, reduced mesenteric lymph nodes, decreased cell numbers, and defects in antibody production relative to conventional animals [58,59,60,61]. Further, the microbiota supports tolerogenic responses and seems to have a role in B cell development [62,63]. Chung *et al.* [53] showed that in mice colonized with mouse gut microbiota (MMb), or human gut microbiota (HMb), bacterial number and phylum abundance in MMb and HMb mice were similar, while the composition of bacterial species in the *Firmicutes* phylum was changed. Moreover, the authors showed that gut immune maturation was dependent on colonization with a host-specific consortium of bacterial species. We could argue that the composition of the commensal microbiota influences individual variations in immunity, and the absence of beneficial host-specific bacteria may promote disease in genetically susceptible individuals [64,65].

Recently Eun *et al.* [66] demonstrated that the colonization of interleukin-10-deficient mice with a simplified human microbiota consortium (SIHUMI), chosen for their relevance to IBD, can induce colonic inflammation and TH1, TH17 mucosal immune responses. The authors also showed that host genetic background and inflammation were able to influence the composition and distribution of intestinal microbial communities and some of its components differentially stimulate murine immune responses. This study highlights the key role of the host in shaping the composition and structure of gut microbiota and adds new elements useful for the understanding the mechanisms that underlie this process. However, the microbiota selected by a specific host genetic background, in turn, will contribute to the maturation and modulation of the immune system as demonstrated recently by several studies [40,66]. Furthermore, the identification of effector strains, within the gut microbial community, that impact immune function will be a challenge for the future.

## 3. Dysbiosis

Dysbiosis is a condition indicating a microbial ecosystem where bacteria do not live in mutual accord, when the “good”, bacteria were not successfully controlling the “bad” ones. Currently, dysbiosis has been linked with important human diseases, including autoimmune and auto inflammatory disorders, such as allergies, obesity, and inflammatory bowel disease, however the genesis of dysbiosis has not yet been clarified. The list of diseases linked to the intestinal microbiota grows every day and these diseases are usually complex in terms of both pathogenesis and complications. Recently the Dysbiosis of Gut Microbiota (DOGMA) was reported to account for all three components of the syndrome of anovulation/menstrual irregularity, hyper-androgenism (acne, hirsutism) and the development of multiple small ovarian cysts [67]. The DOGMA could lead to an increase in gut mucosal permeability, resulting in an increasing passage of lipopolysaccharide (LPS) into the systemic circulation. The subsequent activation of the immune system interferes with insulin receptor function, driving up serum insulin levels, which in turn will increase ovary production of androgens, interfering with normal follicle development [68]. Additionally even in patients with type 2 diabetes a moderate degree of gut microbial dysbiosis was found, such as a decrease in the abundance of butyrate-producing bacteria, an increase in opportunistic pathogens, and an expansion of the microbial functions conferring sulphate reduction and oxidative stress resistance [68]. Among the several hypothesis made recently, lifestyle seems to have a strong influence. In Western countries, where chronic diseases afflict >50% of the adult population the diet, based on elevated consumption of red meat, animal fat, high sugar and low fiber foods, along with the therapeutic use of antibiotics and with a sedentary lifestyle, can play a pivotal role in shaping the microbiota of the human gut. Moreover, it has been demonstrated that the Western diet, induces dysbiosis and contributes to endotoxaemia, most likely caused by impairment of intestinal permeability and barrier function [69,70,71]. We may assume that a high content of fat and sugars in the diet could be a disturbance factor contributing to diseases in genetically susceptible hosts. A recent study reported that the Mediterranean-inspired diet appears to have a benefit in Crohn’s disease patients [72,73]. Several other disturbance factors, such as antibiotic use, affecting the composition of the microbial community, could decrease natural defense mechanisms and cause bacterial overgrowth of potential pathogens species, which may cause serious infections [8,74,75,76,77,78].

### 3.1. Dysbiosis in Inflammatory Bowel Disease (IBD)

An emerging consensus hypothesis is that intestinal dysbiosis may be involved in the pathogenesis of inflammatory bowel disease (IBD), encompassing Crohn’s disease (CD) and ulcerative colitis (UC). IBD are immune-mediated disorders that originate from a breakdown of the normal symbiosis between the mucosal immune system and the commensal microbiota [79,80]. This leads to the development of aberrant reactivity against intraluminal antigens, to dysregulation of the innate and adaptive immunity, and to subsequent tissue injury [81,82]. Several factors could contribute to the loss of tolerance towards some of the indigenous microbiota in patients with IBD, including genetic susceptibility [83], defects in mucosal barrier function [50] and imbalance in the composition of the gut microbiota [9]. In CD, over 71 susceptibility loci have been identified [84,85,86]. Many of the identified gene products are involved in the recognition and processing of microbial antigens at the mucosal surface. The main genetic associations with CD, are the polymorphisms in the nucleotide-binding oligomerization domain-containing protein 2 (NOD2) [87] and in two autophagy-related genes ATG16L1 and IRGM [88,89,90]. Similarly, in UC (ulcerative colitis), genome-wide association studies (GWAS) have identified a total of 47 susceptibility loci, mutations in ECM1 (extracellular matrix protein 1) and amino acid variation on position 11 of HLA-DRβ1, which are specific to patients with UC [91,92]. In CD Th1 lymphocytes, characterized by elevated production of IL2, IL12 and IFNγ, are predominant [92] and in UC, although it has been traditionally considered a Th2-mediated condition, the central role of IL-13, TNF-like cytokine (TL1A), IL-33 and their receptors was recently recognized [93,94]. As suggested by recent evidence, a novel effector pathway, the most prominent of which is the interleukin-23/Th17 axis [95], can mediate tissue injury in inflammatory bowel disease.

A malfunctioning of the endoplasmic reticulum (ER) stress response is also related to IBD patients. Rolhion *et al.* [96] and Kaser *et al.* [97] showed in CD and UC patients, single nucleotide polymorphisms within the XBP1 gene encoding the transcription factor XBP1, a key component of the ER stress response.

It is now well recognized that unresolved ER stress, as a consequence of genetic abnormalities in the unfolded protein response or from a variety of secondary (inflammatory and environmental) factors, leads to cell dysfunction. Taken together, these studies reinforce the idea that ER stress is involved in IBD physiopathology.

An increased intestinal permeability, resulting in a malfunctioning of the epithelial barrier function, is seen in IBD patients. As reported by Salim *et al.* and Schulzke *et al.* [98,99], “Barrier dysfunction, characterized by increased secretion (paracellular and transcellular), of chloride and water (leading to diarrhea) has occurred, with an increased apoptosis of epithelial cells too”. A significant increase in intestinal permeability was reported for 36% of CD patients studied [100] mainly in those carrying NOD2 polymorphisms [101,102]. Inflamed intestinal segments showed a reduction of tight junction fibers, with ruptures, and alterations in the protein content and composition. In early stages of disease, epithelial micro-erosions are the consequence of an up-regulated epithelial apoptosis process, and an increased production of claudin-2. Disease progression could be enhanced by barrier dysfunction and treatments pointed to reestablish the barrier function might offer an alternative or supplement to immunologic-based therapies [103]. Dysbiosis or an imbalance in the GI microbiota of IBD patients has been reported by many authors in numerous studies [9,81,103,104,105]. Frank and coworkers [20], showed that a subset of IBD patients harbor abnormal enteric microbiota, characterized by a reduced proportional abundance of 16S rRNA gene sequencing associated with *Firmicutes* and *Bacteroidetes*, and a concomitant increase in 16S rRNA gene sequencing of *Proteobacteria* and *Actinobacteria*. Among *Firmicutes*, *F. prausnitzii* appears to be particularly under-represented in IBD patients and the reduction in the proportion of this bacterium in intestinal samples was associated with a reduced protection of the gut mucosa [106]. Recently, it has been reported by Duboc and collaborators [107] that IBD-associated dysbiosis was characterized by a decrease in the ratio between *F. prausnitzii* and *E. coli*. To gain insight into the functional consequences of IBD-associated dysbiosis, Morgan *et al.* [108] showed that two phylotypes, *Roseburia*, which are butyrate producers and *Phascolarctobacterium*, which are propionate producers, were significantly reduced in both UC and CD. Particularly in CD patients with ileal involvement, a dramatic reduction in sequences belonging to the *Ruminococcaceae* and *Faecalibacterium* families has been shown [108]. *Roseburia* genera and the *Ruminococcaceae* family are functionally connected with the consumption of hydrogen and with acetate production, that can be utilized by *Roseburia* strains to produce butyrate [108]. *F. prausnitzii*, known to be a butyrate producer, is also able to metabolize polysaccharides derived from the diet and host-derived substrates, like *N*-acetyl glucosamine from intestinal mucus [109].

Many authors have tried to involve specific bacterial species in the onset and/or perpetuation of inflammation, but there is still no compelling evidence that one specific microbe is the etiological agent. *Mycobacterium avium* subsp. *paratuberculosis* is one of the bacterial species proposed to be involved in the onset and/or perpetuation of inflammation, but its role is still debated [110,111].

The finding of exogenous pathogens in IBD patients, reported by several studies, could be explained by the fault in barrier function *versus* the exogenous pathogens, usually exerted by a healthy intestinal microbiota. The barrier malfunctions, together with the loss of “colonization resistance” when dysbiosis status occurs, will expose IBD patients to an increased risk of infections sustained by exogenous infective agents. Almost all studies concerning the characterization of the gut microbiota in IBD patients, showed a significant increase in abundance of bacterial species belonging to the *Enterobacteriaceae* family, specifically *Escherichia/Shigella* [112]. *E. coli* is the most abundant facultative anaerobic bacteria in the intestinal flora of mammals, even if it represents only a minor fraction of the ecosystem (10^5^–10^8^ colony-forming units per gram) [113] in respect to the obligate anaerobic bacteria. This bacterium is a highly adaptable species that can explode in an unbalanced ecosystem [113]. What facilitates its growth in the inflamed intestinal habitat could be due to: its short doubling time; its highly flexibility in metabolic capacity; and its multitude of catabolic pathways. Furthermore, it has recently been reported that the presence of nitric oxide, as a byproduct of the inflammatory habitat, confers a benefit in the growth of *Enterobacteriaceae* such as *E. coli* in the large intestine of mice. The facultative anaerobic bacteria, unlike the strictly anaerobic, may use nitric oxide as a terminal acceptor in cellular anaerobic respiration [114]. The high variability in genomic content of *E. coli* strains, which reflects the high diversity between strains, represents a key factor explaining the selective overgrowth of subtypes whose genomes encode the capability of utilizing inflammation-derived “nutrients” [115].

Darfeuille-Michaud *et al.* [116] observed in a study conducted in 1998 that “*E. coli* strains with adhesive and invasive properties colonized the ileal mucosa of CD patients more frequently than that of control subjects”. The authors further characterized these strains and proposed a potentially new *E. coli* pathovar associated with CD, named adherent-invasive *E. coli* (AIEC) [117]. AIEC strain presence in CD patients is increasing acknowledged; several independent studies from different countries have reported a higher prevalence of *E. coli* AIEC strains in CD patients [112,118,119,120,121]. In the past decade, there have been an increasing number of observations involving adherent-invasive *E. coli* strains (AIEC) in CD pathogenesis, supporting the hypothesis that this pathovar might play an important role in the disease [112,118,119,120,121,122]. Immunological studies report the presence of *E. coli* antigens into macrophage cells within the lamina propria in the mesenteric lymph node centers, in granulomas, and in ulcers of CD patients [123]. CD-involved ileum seems to be an advantageous environment for establishment of a particular *E. coli* genotype, which may have repercussions for IBD progression [124]. An inflamed ileum may furnish a specialized niche permissive for microbes with enhanced fitness in inflamed conditions. Factors favoring *E. coli* intestinal ileum colonization, in CD patients may be: (i) an over expression of CAECAM6 receptor on the apical side of ileal epithelium, through proinflammatory cytokines [125], used by AIEC strains to colonize intestinal tissue via AIEC FimH, and; (ii) an increased expression of the ER stress chaperone Gp96 in the ileal epithelium of CD patients, acting as receptor for the outer membrane vesicles (OMVs) important for the AIEC invasion [97]. These two cellular receptors, co-localizing at the apical surfaces of the ileal epithelium cells, will improve the selective colonization, invasion and persistence of the *E. coli* AIEC. Finally, the ability of such strains to survive and replicate within macrophage cells, further increase the *E. coli* AIEC positive selection. Others factors favoring invasion and persistence of AIEC strains in CD patients could be linked to NOD2, CARD15 and ATG16L1 and IRGM gene mutations, found in many CD patients [88,89,90]. These issues may be involved in the ineffective elimination of bacterial antigens, leading to a progressive over-stimulation of the immune response, a chronic inflammation or uncontrolled inflammation. Recently Chassaing *et al.* [126] showed that the ability of AIEC strains to interact with Peyer’s Patches (PP) and to translocate across the M cells involved an adhesin named long polar fimbriae (LPF). AIEC strains overexpress LPF when there is a high concentration of biliary salt bile in the culture medium. Malabsorption of bile salts predisposes to the formation of the oxalate stones, and is one of the pathological consequences that occur in patients with CD. Although the relative abundance of *E. coli* AIEC strains is significantly higher in IBD patients, these strains were also isolated from healthy subjects. Martinez-Medina *et al.* [118] have highlighted the presence of *E. coli* AIEC strains in 51.9% of CD patients *vs.* 16.7% of control healthy subjects. This gives rise to the idea that *E. coli* AIEC strains are “genetic combinations” favored in “IBD microenvironments”, nevertheless present in healthy subjects too, comparable to the strains named “pathobiontes” [127], strains with pathogenic potential, which under certain conditions can be supported and cause disease. These endogenous strains rarely cause disease except in immune-compromised patients in which the normal gastrointestinal barriers are violated. Certainly DNA acquisition, through horizontal transfers, mediated by plasmid, transposons and phages, played an important role in the transition from commensal to pathogen lifestyle [128]. Nevertheless genomes analysis of the AIEC strain LF82 showed that an important role, in the transition process, is also given to those mutations named pathoadaptive mutations, because they are related to a commensal trait, such as the ones reported in the *fim*H and *omp*A genes present in the LF82 strains [129,130]. Recently AIEC strains have shown the ability to trigger chronic inflammation in genetically susceptible hosts, opening new scenarios on the contribution of such strains to disease [131].

### 3.2. Crohn’s Disease in Pediatric Patients

Crohn’s Colitis Foundation of America (CCFA) defined, as a research priority, the study of disease in pediatric patients. The study of disease in children represents a unique model, where the mechanisms involved in its development are poorly confused or influenced by environmental factors. In contrast with previous studies carried out in adult CD patients, a recent study by Hansen and colleagues [132], reported an increase of the bacterial species, *F. prausnitzii* in pediatric CD patients at disease onset, suggesting a more complex and integral role for *F. prausnitzii* in CD pathogenesis. These apparently conflicting results could find an explanation. As suggested by Hansen and colleagues [132] the higher *F. prausnitzii* colonization of the intestinal habitat observed in pediatric CD patients could represent the early host/microbiota response to CD, with the induction of *F. prausnitzii* proliferation an attempt to reverse the inflammatory change, in view of its multiple anti-inflammatory effects. We could add that in adult CD patients, under circumstances of disease persistence, the intestinal habitat conditions could dramatically change. In fact in adult CD patients’ mucosa, thiol depletion, oxidative stress, bile acid (BA) dysmetabolism, and modifications in pH value and in local oxygen gradients were observed, factors that strongly influenced *F. prausnitzii* growth [132]. In order to evaluate the steps involved in the transition between commensal to pathogen style of life, our group has conducted studies aimed at the characterization of mucosa associated *E. coli* strains isolated from CD pediatric patients [133]. In pediatric patients, the presence of an intestinal dysbiosis, with an increase of mucosa associated bacterial load, especially bacteria belonging to *Enterobacteriaceae* family, mostly *E. coli* it has been reported [133]. In a recently study we found that some *E.coli* FimH variants seem to be more involved in the development of IBD pathogenesis. Moreover several of such variants seems to be related to the Pediatric Crohn’s Disease Activity Index (PCDAI), indicating how the mutagenesis of *fim*H gene responds to specific stimuli/environmental stress, via transitions and/or transversions, differently in different ranges of inflammation [130]. It is possible that AIEC strains could represent an *E. coli* sub-population (pathobionts) with “genetic combinations” that through evolutionary processes could, from commensal phenotypes, reach pathogenic phenotypes [128]. Such genetic variants will strongly contribute to intestinal dysbiosis under inflamed conditions.

### 3.3. Mucosa Associated Microbiota

Special attention should be given to the indigenous mucosa-associated microbiota. The mucosal intestinal area is believed to play an important role in the maintenance of intestinal homeostasis, due to the close proximity to the intestinal epithelium, and to the underlying mucosal immune system. In healthy subjects the intestinal epithelium is not strongly colonized, indicating a strict colonization control. Our group has recently published a study demonstrating that the intestinal mucosa of patients with IBD and Celiac patients is poorly colonized by the bacterial predator named *Bdellovibrio bacteriovorus* when compared with the mucosa of healthy subjects [134]. The low prevalence of *B. bacteriovorus* at the mucosal level, in both IBD and Celiac patients, could support the idea that a loss in microbiota biodiversity could also involve species, such as predatory *Bdellovibrio*, that function in regulating the bacterial population levels, keeping mucosa colonization under strict control. This finding needs to be confirmed in a larger cohort.

## 4. Conclusions

**Figure 1:**
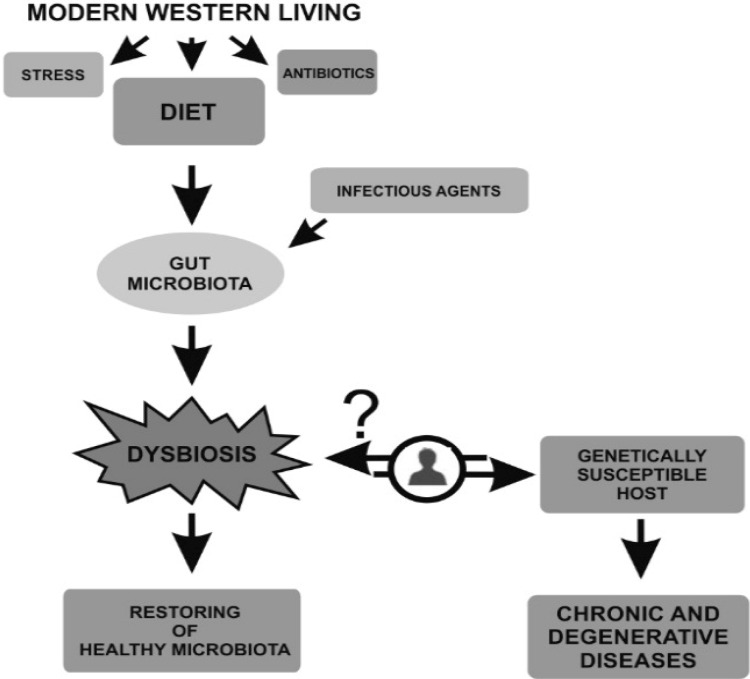
Triggering factors dysbiosis.

In conclusion, it is clear that changes in the structure and composition of the commensal microbiota have an impact on human health. The original question: “Is dysbiosis simply a consequence of chronic inflammation, or a primary trigger that leads to pathogenesis?” is still open. Based on the literature, modern Western lifestyle and infectious agents [69,135] are considered the most important triggering factors for gut dysbiosis (Figure 1). In genetically susceptible hosts, the changes in the gut microbiota composition could have a profound impact on chronic disease development. As recently reported by Eun *et al.* [66], the host genetic background has a strong influence in shaping gut microbiota composition. The authors showed, for the first time, that a distinct human bacterial association is able to induce inflammation in genetically susceptible gnotobiotic mice. To date, therefore, the answer to the question is still far from clear. We believe that in IBD, and related multifactorial pathologies, genetic susceptibility and gut dysbiosis are intimately correlated, but not sufficient to generate disease individually.

## References

[1] Sekirov I., Russell S.L., Antunes L.C., Finlay B.B. (2010). Gut microbiota in health and disease. Physiol. Rev..

[2] Bik E.M. (2009). Composition and function of the human-associated microbiota. Nutr. Rev..

[3] Hawrelak J.A., Myers S.P. (2004). The causes of intestinal dysbiosis. Altern. Med. Rev..

[4] Ley R.E., Backhed F., Turnbaugh P., Lozupone C.A., Knight R.D., Gordon J.I. (2005). Obesity alters gut microbial ecology. Proc. Natl. Acad. Sci. USA.

[5] Cani P.D., Bibiloni R., Knauf C., Waget A., Neyrinck A.M., Delzenne N.M., Burcelin R. (2008). Changes in gut microbiota control metabolic endotoxemia-induced inflammation in high-fat diet-induced obesity and diabetes in mice. Diabetes.

[6] Noverr M.C., Huffnagle G.B. (2005). The “microflora hypothesis” of allergic diseases. Clin. Exp. Allergy.

[7] Frank D.N., Zhu W., Sartor R.B., Li E. (2011). Investigating the biological and clinical significance of human dysbioses. Trends Microbiol..

[8] Sullivan A., Edlund C., Nord C.E. (2001). Effect of antimicrobial agents on the ecological balance of human microflora. Lancet Infect. Dis..

[9] Tamboli C.P., Neut C., Desreumaux P., Colombel J.F. (2004). Dysbiosis in inflammatory bowel disease. Gut.

[10] Sartor R.B. (2008). Microbial influences in inflammatory bowel diseases. Gastroenterology.

[11] Ley R.E., Peterson D.A., Gordon J.I. (2006). Ecological and evolutionary forces shaping microbial diversity in the human intestine. Cell.

[12] Chow J., Lee S.M., Shen Y., Khosravi A., Mazmanian S.K. (2010). Host-bacterial symbiosis in health and disease. Adv. Immunol..

[13] Eckburg P.B., Bik E.M., Bernstein C.N., Purdom E., Dethlefsen L., Sargent M., Gill S.R., Nelson K.E., Relman D.A. (2005). Diversity of the human intestinalmicrobial flora. Science.

[14] Turnbaugh P.J., Ley R.E., Hamady M., Fraser-Liggett C.M., Knight R., Gordon J.I. (2007). The human microbiome project. Nature.

[15] Li J., Jia H., Cai X., Zhong H., Feng Q., Sunagawa S., Arumugam M., Kultima J.R., Prifti E., Nielsen T. (2014). An integrated catalog of reference genes in the human gut microbiome. Nat. Biotechnol..

[16] O’Hara A.M., Shanahan F. (2006). The gut flora as a forgotten organ. EMBO Rep..

[17] Berg R.D. (1996). The indigenous gastrointestinal microflora. Trends Microbiol..

[18] Sommer F., Bäckhed F. (2013). The gut microbiota masters of host development and physiology. Nat. Rev. Microbiol..

[19] Korecka A., Arulampalam V. (2012). The gut microbiome: Scourge, sentinel or spectator?. J. Oral Microbiol..

[20] Frank D.N., Amand A.L., Feldman R.A., Boedeker E.C., Harpaz N., Pace N.R. (2007). Molecular-phylogenetic characterization of microbial community imbalances in human inflammatory bowel diseases. Proc. Natl. Acad. Sci. USA.

[21] Turnbaugh P.J., Gordon J.I. (2009). The core gut microbiome, energy balance and obesity. J. Physiol..

[22] Qin J., Li R., Raes J., Arumugam M., Burgdorf K.S., Manichanh C., Nielsen T., Pons N., Levenez F., Yamada T. (2010). Human gut microbial gene catalogue established by metagenomic sequencing. Nature.

[23] Arumugam M., Raes J., Pelletier E., le Paslier D., Yamada T., Mende D.R., Fernandes G.R., Tap J., Bruls T., Batto J.M. (2011). Enterotypes of the human gut microbiome. Nature.

[24] Ed Yong Gut Microbial “Enterotypes” Become Less Clear-Cut. Communities of Gut Bacteria May Form a Spectrum Rather than Falling into Distinct Groups. http://www.nature.com/news/gut-microbial-enterotypes-become-less-clear-cut-1.10276.

[25] Scanlan P.D., Shanahan F., Marchesi J.R. (2008). Human methanogen diversity and incidence in healthy and diseased colonic groups using mcrA gene analysis. BMC Microbiol..

[26] Yachi S., Loreau M. (1999). Biodiversity and ecosystem productivity in a fluctuating environment: The insurance hypothesis. Proc. Natl. Acad. Sci. USA.

[27] Tremaroli V., Bäckhed F. (2012). Functional interactions between the gut microbiota and host metabolism. Nature.

[28] Macfarlane S., Macfarlane G.T. (2003). Regulation of short-chain fatty acid production. Proc. Nutr. Soc..

[29] Falony G., Vlachou A., Verbrugghe K., de Vuyst L. (2006). Cross feeding between *Bifidobacterium longum* BB536 and acetate converting, butyrate-producing colon bacteria during growth on oligo fructose. Appl. Environ. Microbiol..

[30] Lefebvre P., Cariou B., Lien F., Kuipers F., Staels B. (2009). Role of bile acids and bile acid receptors in metabolic regulation. Physiol. Rev..

[31] Li M., Wang B., Zhang M., Rantalainen M., Wang S., Zhou H., Zhang Y., Shen J., Pang X., Zhang M. (2008). Symbiotic gut microbes modulate human metabolic phenotypes. PNAS.

[32] Mai V. (2004). Dietary modification of the intestinal microbiota. Nutr. Rev..

[33] Lunn J.C., Kuhnle G., Mai V., Frankenfeld C., Shuker D.E., Glen R.C., Goodman J.M., Pollock J.R., Bingham S.A. (2007). The effect of haem in red and processed meat on the endogenous format ion of *N*-nitroso compounds in the upper gastrointestinal tract. Carcinogenesis.

[34] Lepage P., Leclerc M.C., Joossens M., Mondot S., Blottière H.M., Raes J., Ehrlich D., Doré J. (2013). A metagenomic insight into our gut’s microbiome. Gut.

[35] Saemann M.D., Bohmig G.A., Zlabinger G.J. (2002). Short-chain fatty acids: Bacterial mediators of a balanced host-microbial relationship in the human gut. Wien. Klin. Wochenschr..

[36] Hamer H.M., Jonkers D., Venema K., Vanhoutvin S., Troost F.J., Brummer R.J. (2008). The role of butyrate on colonic function. Aliment. Pharmacol. Ther..

[37] Berndt B.E., Zhang M., Owyang S.Y., Cole T.S., Wang T.W., Luther J., Veniaminova N.A., Merchant J.L., Chen C.C., Huffnagle G.B. (2012). Butyrate increases IL-23 production by stimulated dendritic cells. Am. J. Physiol. Gastrointest. Liver. Physiol..

[38] Furusawa Y., Obata Y., Fukuda S., Endo T.A., Nakato G., Takahashi D., Nakanishi Y., Uetake C., Kato K., Kato T. (2013). Commensal microbe-derived butyrate induces the differentiation of colonic regulatory T cells. Nature.

[39] Arpaia N., Campbell C., Fan X., Dikiy S., van der Veeken J., deRoos P., Liu H., Cross J.R., Pfeffer K., Coffer P.J. (2013). Metabolites produced by commensal bacteria promote peripheral regulatory T-cell generation. Nature.

[40] Atarashi K., Tanoue T., Oshima K., Suda W., Nagano Y., Nishikawa H., Fukuda S., Saito T., Narushima S., Hase K. (2013). Treg induction by a rationally selected mixture of *Clostridia* strains from the human microbiota. Nature.

[41] Sousa T., Paterson R., Moore V., Carlsson A., Abrahamsson B., Basit A.W. (2008). The gastrointestinal microbiota as a site for the biotransformation of drugs. Int. J. Pharm..

[42] Florin T., Neale G., Gibson G.R., Christl S.U., Cummings J.H. (1991). Metabolism of dietary sulphate: Absorption and excretion in humans. Gut.

[43] Petersen L.C. (1977). The effect of inhibitors on the oxygen kinetics of cytochrome c oxidase. Biochim. Biophys. Acta.

[44] Vermeiren J., Hindryckx P., van Nieuwenhuyse G., Laukens D., de Vos M., Boon N., van de Wiele T. (2012). Intrarectal nitric oxide administration prevents cellular infiltration but not colonic injury during dextran sodium sulfate colitis. Dig. Dis. Sci..

[45] Chung H., Kasper D.L. (2010). Microbiota-stimulated immune mechanisms to maintain gut homeostasis. Curr. Opin. Immunol..

[46] Savage D.J., Siegel J., Snellen D.W. (1981). Transit time of epithelial cells in the small intestines of germ-free mice and ex-germfree mice associated with indigenous microorganisms. Appl. Environ. Microbiol..

[47] Shirkey T.W., Siggers R.H., Goldade B.G., Marshall J.K., Drew M.D., Laarveld B., van Kessel A.G. (2006). Effects of commensal bacteria on intestinal morphology and expression of proin-flammatory cytokines in the gnotobiotic pig. Exp. Biol. Med..

[48] Hooper L.V., Wong M.H., Thelin A., Hansson L., Falk P.G., Gordon J.I. (2001). Molecular analysis of commensal host-microbial relationships in the intestine. Science.

[49] Cario E., Gerken G., Podolsky D.K. (2007). Toll-like receptor 2 controls mucosal inflammation by regulating epithelial barrier function. Gastroenterology.

[50] Rakoff-Nahoum S., Paglino J., Eslami-Varzaneh F., Edberg S., Medzhitov R. (2004). Recognition of commensal microflora by toll-like receptors is required for intestinal homeostasis. Cell.

[51] Leatham M.P., Banerjee S., Autieri S.M., Mercado-Lubo R., Conway T., Cohen P.S. (2009). Precolonized human commensal *Escherichia coli* strains serve as a barrier to *E. coli* O157: H7 growth in the streptomycin-treated mouse intestine. Infect. Immun..

[52] Van der Waaij D., Berghuis-de Vries J.M., Lekkerkerk V. (1971). Colonization resistance of the digestive tract in conventional and antibiotic-treated mice. J. Hyg. (Lond.).

[53] Chung H., Pamp S.J., Hill J.A., Surana N.K., Edelman S.M., Troy E.B., Reading N.C., Villablanca E.J., Wang S., Mora J.R. (2012). Gut immune maturation depends on colonization with a host-specific microbiota. Cell.

[54] Vaishnava S., Behrendt C.L., Ismail A.S., Eckmann L., Hooper L.V. (2008). Paneth cells directly sense gut commensals and maintain homeostasis at the intestinal host-microbial interface. Proc. Natl. Acad. Sci. USA.

[55] Wrzosek L., Miquel S., Noordine M.L., Bouet S., Joncquel Chevalier-Curt M., Robert V., Philippe C., Bridonneau C., Cherbuy C., Robbe-Masselot C. (2013). *Bacteroides thetaiotaomicron* and *Faecalibacterium prausnitzii* influence the production of mucus glycans and the development of goblet cells in the colonic epithelium of a gnotobiotic model rodent. BMC Biol..

[56] Hill D.A., Hoffmann C., Abt M.C., Du Y., Kobuley D., Kirn T.J., Bushman F.D., Artis D. (2010). Metagenomic analyses reveal antibiotic-induced temporal and spatial changes in intestinal microbiota with associated alterations in immune cell homeostasis. Mucosal Immunol..

[57] Macpherson A.J., Harris N.L. (2004). Interactions between commensal intestinal bacteria and the immune system. Nat. Rev. Immunol..

[58] Round J.L., Mazmanian S.K. (2009). The gut microbiota shapes intestinal immune responses during health and disease. Nat. Rev. Immunol..

[59] Reis B.S., Mucida D. (2012). The role of the intestinal context in the generation of tolerance and inflammation. Clin. Dev. Immunol..

[60] Lee Y.K., Mazmanian S.K. (2010). Has the microbiota played a critical role in the evolution of the adaptive immune system?. Science.

[61] Haverson K., Rehakova Z., Sinkora J., Sver L., Bailey M. (2007). Immune development in jejunal mucosa after colonization with selected commensal gut bacteria: A study in germ-free pigs. Vet. Immunol. Immunopathol..

[62] O’Mahony C., Scully P., O’Mahony D., Murphy S., O’Brien F., Lyons A., Sherlock G., MacSharry J., Kiely B., Shanahan F. (2008). Commensal-induced regulatory T cells mediate protection against pathogen-stimulated NF-kappaB activation. PLoS Pathog..

[63] Gaboriau-Routhiau V., Rakotobe S., Lécuyer E., Mulder I., Lan A., Bridonneau C., Rochet V., Pisi A., de Paepe M., Brandi G. (2009). The key role of segmented filamentous bacteria in the coordinated maturation of gut helper T cell responses. Immunity.

[64] Wills-Karp M., Santeliz J., Karp C.L. (2001). The germless theory of allergic disease: Revisiting the hygiene hypothesis. Nat. Rev. Immunol..

[65] Wu H.J., Ivanov I.I., Darce J., Hattori K., Shima T., Umesaki Y., Littman D.R., Benoist C., Mathis D. (2010). Gut-residing segmented filamentous bacteria drive autoimmune arthritis via T helper 17 cells. Immunity.

[66] Eun C.S., Mishima Y., Wohlgemuth S., Liu B., Bower M., Carroll I.M., Sartor R.B. (2014). Induction of bacterial antigen-specific colitis by a simplified human microbiota consortium in gnotobiotic interleukin-10/mice. Infect Immun..

[67] Tremellen K., Pearce K. (2012). Dysbiosis of Gut Microbiota (DOGMA)—A novel theory for the development of Polycystic Ovarian Syndrome. Med. Hypotheses.

[68] Qin J., Li Y., Cai Z., Li S., Zhu J., Zhang F., Liang S., Zhang W., Guan Y., Shen D. (2012). A metagenome-wide association study of gut microbiota in type 2 diabetes. Nature.

[69] Martinez-Medina M., Denizot J., Dreux N., Robin F., Billard E., Bonnet R., Darfeuille-Michaud A., Barnich N. (2014). Western diet induces dysbiosis with increased *E. coli* in CEABAC10 mice, alters host barrier function favouring AIEC colonisation. Gut.

[70] Huang E.Y., Devkota S., Moscoso D., Chang E.B., Leone V.A. (2013). The role of diet in triggering human inflammatory disorders in the modern age. Microbes Infect..

[71] Pendyala S., Walker J.M., Holt P.R. (2012). A high-fat diet is associated with endotoxemia that originates from the gut. Gastroenterology.

[72] Marlow G., Ellett S., Ferguson I.R., Zhu S., Karunasinghe N., Jesuthasan A.C., Han D.Y., Fraser A.G., Ferguson L.R. (2013). Transcriptomics to study the effect of a Mediterranean-inspired diet on inflammation in Crohn’s disease patients. Hum. Genomics.

[73] Nord C.E. (1993). The effect of antimicrobial agents on the ecology of the human intestinal microflora. Vet. Microbiol..

[74] Gismondo M.R. (1998). Antibiotic impact on intestinal microflora. Gastroenterol. Int..

[75] Muegge B.D., Kuczynski J., Knights D., Clemente J.C., González A., Fontana L., Henrissat B., Knight R., Gordon J.I. (2011). Diet drives convergence in gut microbiome functions across mammalian phylogeny and within humans. Science.

[76] Rafii F., Sutherland J.B., Cerniglia C.E. (2008). Effects of treatment with antimicrobial agents on the human colonic microflora. Ther. Clin. Risk Manag..

[77] Hurley B.W., Nguyen C.C. (2002). The spectrum of pseudomembranous enterocolitis and antibiotic-associated diarrhea. Arch. Intern. Med..

[78] Xavier R.J., Podolsky D.K. (2007). Unraveling the pathogenesis of inflammatory bowel disease. Nature.

[79] Podolsky D.K. (1997). Lessons from genetic models of inflammatory bowel disease. Acta Gastroenterol. Belg..

[80] Sartor R.B. (1997). Pathogenesis and immune mechanisms of chronic inflammatory bowel diseases. Am. J. Gastroenterol..

[81] Cho J.H. (2008). The genetics and immunopathogenesis of inflammatory bowel disease. Nat. Rev. Immunol..

[82] Lees C.W., Barrett J.C., Parkes M., Satsangi J. (2011). New IBD genetics: Common pathways with other diseases. Gut.

[83] Rioux J.D., Xavier R.J., Taylor K.D., Silverberg M.S., Goyette P., Huett A., Green T., Kuballa P., Barmada M.M., Datta L.W. (2007). Genome-wide association study identifies new susceptibility loci for Crohn disease and implicates autophagy in disease pathogenesis. Nat. Genet..

[84] Walker A.W., Sanderson J.D., Churcher C., Parkes G.C., Hudspith B.N., Rayment N., Brostoff J., Parkhill J., Dougan G., Petrovska L. (2011). High-throughput clone library analysis of the mucosa-associated microbiota reveals dysbiosis and differences between inflamed and non-inflamed regions of the intestine in inflammatory bowel disease. BMC Microbiol..

[85] Rivas M.A., Beaudoin M., Gardet A., Stevens C., Sharma Y., Zhang C.K., Boucher G., Ripke S., Ellinghaus D., Burtt N. (2011). Deep resequencing of GWAS loci identifies independent rare variants associated with inflammatory bowel disease. Nat. Genet..

[86] Inohara N., Ogura Y., Fontalba A., Gutierrez O., Pons F., Crespo J., Fukase K., Inamura S., Kusumoto S., Hashimoto M. (2003). Host recognition of bacterial muramyl dipeptide mediated through NOD2. Implications for Crohn’s disease. J. Biol. Chem..

[87] Hugot J.P., Chamaillard M., Zouali H., Lesage S., Cézard J.P., Belaiche J., Almer S., Tysk C., O’Morain C.A., Gassull M. (2001). Association of NOD2 leucine-rich repeat variants with susceptibility to Crohn’s disease. Nature.

[88] Hampe J., Franke A., Rosenstiel P., Till A., Teuber M., Huse K., Albrecht M., Mayr G., de la Vega F.M., Briggs J. (2007). A genome-wide association scan of nonsynonymous SNPs identifies a susceptibility variant for Crohn disease in ATG16L1. Nat. Genet..

[89] Parkes M., Barrett J.C., Prescott N.J., Tremelling M., Anderson C.A., Fisher S.A., Roberts R.G., Nimmo E.R., Cummings F.R., Soars D. (2007). Sequence variants in the autophagy gene IRGM and multiple other replicating loci contribute to Crohn’s disease susceptibility. Nat. Genet..

[90] Travassos L.H., Carneiro L.A., Ramjeet M., Hussey S., Kim Y.G., Magalhães J.G., Yuan L., Soares F., Chea E., le Bourhis L. (2010). Nod1 and Nod2 direct autophagy by recruiting ATG16L1 to the plasma membrane at the site of bacterial entry. Nat. Immunol..

[91] Achkar J.P., Klei L., de Bakker P.I., Bellone G., Rebert N., Scott R., Lu Y., Regueiro M., Brzezinski A., Kamboh M.I. (2012). Amino acid position 11 of HLA-DRβ1 is a major determinant of chromosome 6p association with ulcerative colitis. Genes Immun..

[92] Bamias G., Cominelli F. (2007). Immunopathogenesis of inflammatory bowel disease: Current concepts. Curr. Opin. Gastroenterol..

[93] Cayrol C., Girard J.P. (2009). The IL-1-like cytokine IL-33 is inactivated after maturation by caspase-1. Proc. Natl. Acad. Sci. USA.

[94] Mannon P.J., Hornung R.L., Yang Z., Yi C., Groden C., Friend J., Yao M., Strober W., Fuss I.J. (2011). Suppression of inflammation in ulcerative colitis by interferon-β-1a is accompanied by inhibition of IL-13 production. Gut.

[95] Sarra M., Pallone F., Macdonald T.T., Monteleone G. (2010). IL-23/IL-17 axis in IBD. Inflamm. Bowel Dis..

[96] Rolhion N., Hofman P., Darfeuille-Michaud A. (2011). The endoplasmic reticulum stress response chaperone: Gp96, a host receptor for Crohn disease-associated adherent-invasive *Escherichia coli*. Gut Microbes.

[97] Kaser A., Lee A.H., Franke A., Glickman J.N., Zeissig S., Tilg H., Nieuwenhuis E.E., Higgins D.E., Schreiber S., Glimcher L.H. (2008). XBP1 links ER stress to intestinal inflammation and confers genetic risk for human inflammatory bowel disease. Cell.

[98] Salim S.Y., Söderholm J.D. (2011). Importance of disrupted intestinal barrier in inflammatory bowel diseases. Inflamm. Bowel Dis..

[99] Schulzke J.D., Bojarski C., Zeissig S., Heller F., Gitter A.H., Fromm M. (2006). Disrupted barrier function through epithelial cell apoptosis. Ann. N. Y. Acad. Sci..

[100] Benjamin J., Makharia G.K., Ahuja V., Kalaivani M., Joshi Y.K. (2008). Intestinal permeability and its association with the patient and disease characteristics in Crohn’s disease. World J. Gastroenterol..

[101] Matricon J. (2010). Immunopathogenesis of inflammatory bowel disease. Med. Sci..

[102] D’Incà R., Annese V., di Leo V., Latiano A., Quaino V., Abazia C., Vettorato M.G., Sturniolo G.C. (2006). Increased intestinal permeability and NOD2 variants in familial and sporadic Crohn’s disease. Aliment. Pharmacol. Ther..

[103] Edelblum K.L., Turner J.R. (2009). The tight junction in inflammatory disease: Communication breakdown. Curr. Opin. Pharmacol..

[104] Ott S.J., Musfeldt M., Wenderoth D.F., Hampe J., Brant O., Fölsch U.R., Timmis K.N., Schreiber S. (2004). Reduction in diversity of the colonic mucosa associated bacterial microflora in patients with active inflammatory bowel disease. Gut.

[105] Manichanh C., Rigottier-Gois L., Bonnaud E., Gloux K., Pelletier E., Frangeul L., Nalin R., Jarrin C., Chardon P., Marteau P. (2006). Reduced diversity of faecal microbiota in Crohn’s disease revealed by a metagenomic approach. Gut.

[106] Sokol H., Seksik P., Furet J.P., Firmesse O., Nion-Larmurier I., Beaugerie L., Cosnes J., Corthier G., Marteau P., Doré J. (2009). Low counts of *Faecalibacterium prausnitzii* in colitis microbiota. Inflamm. Bowel Dis..

[107] Duboc H., Rajca S., Rainteau D., Benarous D., Maubert M.A., Quervain E., Thomas G., Barbu V., Humbert L., Despras G. (2013). Connecting dysbiosis, bile-acid dysmetabolism and gut inflammation in inflammatory bowel diseases. Gut.

[108] Morgan X.C., Tickle T.L., Sokol H., Gevers D., Devaney K.L., Ward D.V., Reyes J.A., Shah S.A., LeLeiko N., Snapper S.B. (2012). Dysfunction of the intestinal microbiome in inflammatory bowel disease and treatment. Genome Biol..

[109] Lopez-Siles M., Khan T.M., Duncan S.H., Harmsen H.J., Garcia-Gil L.J., Flint H.J. (2012). Cultured representatives of two major phylogroups of human colonic *Faecalibacterium prausnitzii* can utilize pectin, uronic acids, and host-derived substrates for growth. Appl. Environ. Microbiol..

[110] El-Zaatari F.A., Naser S.A., Hulten K., Burch P., Graham D.Y. (1999). Characterization of *Mycobacterium paratuberculosis* p36 antigen and its seroreactivities in Crohn’s disease. Curr. Microbiol..

[111] Naser S.A., Ghobrial G., Romero C., Valentine J.F. (2004). Culture of *Mycobacterium avium* subspecies paratuberculosis from the blood of patients with Crohn’s disease. Lancet.

[112] Baumgart M., Dogan B., Rishniw M., Weitzman G., Bosworth B., Yantiss R., Orsi R.H., Wiedmann M., McDonough P., Kim S.G. (2007). Culture independent analysis of ileal mucosa reveals a selective increase in invasive *Escherichia coli* of novel phylogeny relative to depletion of *Clostridiales* in Crohn’s disease involving the ileum. ISME J..

[113] Stecher B., Maier L., Hardt W.D. (2013). “Blooming” in the gut: How dysbiosis might contribute to pathogen evolution. Nat. Rev. Microbiol..

[114] Winter S.E., Winter M.G., Xavier M.N., Thiennimitr P., Poon V., Keestra A.M., Laughlin R.C., Gomez G., Wu J., Lawhon S.D. (2013). Host-derived nitrate boosts growth of *E. coli* in the inflamed gut. Science.

[115] Winter S.E., Bäumler A.J. (2014). Dysbiosis in the inflamed intestine: Chance favors the prepared microbe. Gut Microbes.

[116] Darfeuille-Michaud A., Neut C., Barnich N., Lederman E., di Martino P., Desreumaux P., Gambiez L., Joly B., Cortot A., Colombel J.F. (1998). Presence of adherent *Escherichia coli* strains in ileal mucosa of patients with Crohn’s disease. Gastroenterology.

[117] Boudeau J., Glasser A.L., Masseret E., Joly B., Darfeuille-Michaud A. (1999). Invasive ability of an *Escherichia coli* strain isolated from the ileal mucosa of a patient with Crohn’s disease. Infect. Immun..

[118] Martinez-Medina M., Aldeguer X., Lopez-Siles M., González-Huix F., López-Oliu C., Dahbi G., Blanco J.E., Blanco J., Garcia-Gil L.J., Darfeuille-Michaud A. (2009). Molecular diversity of *Escherichia coli* in the human gut: New ecological evidence supporting the role of adherent-invasive *E. coli* (AIEC) in Crohn’s disease. Inflamm. Bowel Dis..

[119] Darfeuille-Michaud A., Boudeau J., Bulois P., Neut C., Glasser A.L., Barnich N.M., Bringer A., Swidsinski A., Beaugerie L., Colombel J.F. (2004). High prevalence of adherent-invasive *Escherichia coli* associated with ileal mucosa in Crohn’s disease. Gastroenterology.

[120] Sasaki M., Sitaraman S.V., Babbin B.A., Gerner-Smidt P., Ribot E.M., Garrett N., Alpern J.A., Akyildiz A., Theiss A.L., Nusrat A. (2007). Invasive *Escherichia coli* are a feature of Crohn’s disease. Lab. Investig..

[121] Glasser A.L., Darfeuille-Michaud A. (2008). Abnormalities in the handling of intracellular bacteria in Crohn’s disease: A link between infectious etiology and host genetic susceptibility. Arch. Immunol. Ther. Exp..

[122] Martin H.M., Campbell B.J., Hart C.A., Mpofu C., Nayar M., Singh R., Englyst H., Williams H.F., Rhodes J.M. (2004). Enhanced *Escherichia coli* adherence and invasion in Crohn’s disease and colon cancer. Gastroenterology.

[123] Ryan P., Kelly R.G., Lee G., Collins J.K., O’Sullivan G.C., O’Connell J., Shanahan F. (2004). Bacterial DNA within granulomas of patients with Crohn’s disease—Detection by laser capture microdissection and PCR. Am. J. Gastroenterol..

[124] Flanagan P., Campbell B.J., Rhodes J.M. (2011). Bacteria in the pathogenesis of inflammatory bowel disease. Biochem. Soc. Trans..

[125] Barnich N., Carvalho F.A., Glasser A.L., Darcha C., Jantscheff P., Allez M., Peeters H., Bommelaer G., Desreumaux P., Colombel J.F. (2007). CEACAM6 acts as a receptor for adherent-invasive *E. coli*, supporting ileal mucosa colonization in Crohn disease. J. Clin. Invest..

[126] Chassaing B., Darfeuille-Michaud A. (2011). The interaction of Crohn’s disease-associated *Escherichia coli* to Peyer’s patches of the intestinal mucosa involves long polar fimbriae. Med. Sci..

[127] Schippa S., Iebba V., Totino V., Santangelo F., Lepanto M., Alessandri C., Nuti F., Viola F., di Nardo G., Cucchiara S. (2012). A potential role of *Escherichia coli* pathobionts in the pathogenesis of pediatric inflammatory bowel disease. Can. J. Microbiol..

[128] Alteri C.J., Mobley H.L. (2012). *Escherichia coli* physiology and metabolism dictates adaptation to diverse host microenvironments. Curr. Opin. Microbiol..

[129] Miquel S., Peyretaillade E., Claret L., de Vallée A., Dossat C., Vacherie B., el Zineb H., Segurens B., Barbe V., Sauvanet P. (2010). Complete genome sequence of Crohn’s disease-associated adherent-invasive *E. coli* strain LF82. PLoS One.

[130] Iebba V., Conte M.P., Lepanto M.S., di Nardo G., Santangelo F., Aloi M., Totino V., Checchi M.P., Longhi C., Cucchiara S. (2012). Microevolution in *fimH* gene of mucosa-associated *Escherichia coli* strains isolated from pediatric patients with inflammatory bowel disease. Infect. Immun..

[131] Chassaing B., Koren O., Carvalho F.A., Ley R.E., Gewirtz A.T. (2014). AIEC pathobiont instigates chronic colitis in susceptible hosts by altering microbiota composition. Gut.

[132] Hansen R., Russell R.K., Reiff C., Louis P., McIntosh F., Berry S.H., Mukhopadhya I., Bisset W.M., Barclay A.R., Bishop J. (2012). Microbiota of *de-novo* pediatric IBD: Increased *Faecalibacterium prausnitzii* and reduced bacterial diversity in Crohn’s but not in ulcerative colitis. Am. J. Gastroenterol..

[133] Conte M.P., Schippa S., Zamboni I., Penta M., Chiarini F., Seganti L., Osborn J., Falconieri P., Borrelli O., Cucchiara S. (2006). Gut-associated bacterial microbiota in paediatric patients with inflammatory bowel disease. Gut.

[134] Iebba V., Santangelo F., Totino V., Nicoletti M., Gagliardi A., de Biase R.V., Cucchiara S., Nencioni L., Conte M.P., Schippa S. (2013). Higher prevalence and abundance of *Bdellovibrio bacteriovorus* in the human gut of healthy subjects. PLoS One.

[135] Lee A., Griffiths T.A., Parab R.S., King R.K., Dubinsky M.C., Urbanski S.J., Wrobel I., Rioux K.P. (2011). Association of *Mycobacterium avium* subspecies paratuberculosis with Crohn Disease in pediatric patients. J. Pediatr. Gastroenterol. Nutr..

